# Anthropometric Predictors of Appropriate-Sized Plastibell for Neonatal Circumcision

**DOI:** 10.4314/ejhs.v33i4.15

**Published:** 2023-07

**Authors:** Victor Ifeanyichukwu Modekwe, Jideofor Okechukwu Ugwu, Okechukwu Hyginus Ekwunife, Charles Chidiebele Maduba, Ugochukwu Uzodimma Nnadozie, Ezekiel Uchechukwu Nwankwo

**Affiliations:** 1 Department of Surgery, Nnamdi Azikiwe University Awka; 2 Paediatric Surgery Unit, Department of Surgery, Nnamdi Azikiwe University Teaching Hospital, Nnewi, Anambra State, Nigeria; 3 Division of Plastic Surgery, Department of Surgery, Alex Ekwueme Federal University Teaching Hospital, Abakaliki, Ebonyi State, Nigeria

**Keywords:** Plastibell circumcision, Neonatal circumcision, penile, penile length

## Abstract

**Background:**

Using anthropometric parameters to determine the appropriate Plastibell size before circumcision ensures that cumbersome carrying of all the sizes before each procedure is eliminated and also complications reduced.

**Methods:**

Male neonates who presented for routine circumcision by Plastibell method were recruited. Collected on a proforma were their age in days, weight in Kg, stretched penile length (SPL) in cm, penile diameter (PD) in cm and the Plastibell size used by the “circumciser”. The routine circumcision was carried out for each neonate according to protocol. P value was set at <0.05.

**Results:**

There were 231 neonates who had Plastibell circumcision. Their mean age, weight, SPL and PD were 15.6(±5.73) days, 3.7(±0.58) Kg, 3.66 (±0.58) cm and 3.79 (±0.64) cm, respectively. Plastibell size 1.3 is the most used (53.6%). There was a positive correlation between weight, SPL, PD, on one hand and Plastibell size , on the other hand with P-values of <0.001, <0.001 and <0.001 respectively. The weight was a weak determinant for Plastibell sizes 1.1 and 1.3: (OR 7.104; 95% CI 1.108 - 45.559; P = .039) and (OR 2.044; 95% CI 1.054 - 3.963; P = .034) respectively. The SPL is also a weak predictor for Plastibell sizes 1.2 and 1.5: (OR 2.176; 95% CI 1.136 -4.136; P = .019) and (OR .043; 95% CI .072 - .984; P = .047), respectively.

**Conclusion:**

The anthropometric parameters correlate well with Plastibell sizes. However, they are not effective in predicting the appropriate sized Plastibell for neonatal circumcision.

## Introduction

Neonatal circumcision is the most common surgical procedure (1). This is in spite of the controversies associated with circumcision (1–3). The procedure has continued to maintain ground and gain acceptability among the populace (1-6). There are various methods of conducting a neonatal circumcision. The occlusive methods of circumcision are adjudged to be the safest of all the methods. The occlusive methods are Plastibell®, Gomco, Mogen, Tara clamp, Zhenxi clamp, etc. Plastibell® method of circumcision is very common in our environment. It has continued to gain ground and acceptance among practitioners and seekers of circumcision. This is because of its safety, simplicity to learn and to conduct. Also the outcome is very good and acceptable to the mothers and seekers of the procedure. It can be used in neonates, infants and younger children (3,4,6).

Plastibell^®^ comes as a transparent ring with a grove around its external circumference (4). It has a bell-handle across its diameter, on one side. The packaging of the Plastibell^®^ comes with a thread. Plastibell ring comes in sizes 1.1 to 1.7 (4,7). These figures correspond directly to the internal diameter of the Plastibell^®^ ring. The procedure involves restraining the child in a lithotomy position. The perineum including the external genitalia is cleansed with a mild antiseptic solution. An anaesthetic is applied depending on the practice of the surgeon, and some choice. The procedure involves four major steps: dorsal slit, adhesiolysis, tying of thread to exsanguinate the prepuce over the Plastibell ring's groove, and excision of excess prepuce (3,4). The third step is protective of the glans which is prone to catastrophic injuries in the circumcision procedure. This is the selling point of Plastibell^®^ circumcision. Again the tying of the thread apart from exsanguinating effect ensures that haemorrhage - primary and secondary are largely prevented (7).

However Plastibell^®^ circumcision has its own peculiar complications, which can constitute a major problem (6-9). These include Plastibell ring retention and proximal migration of the ring (6). These problems result from inappropriate choice of and application of Plastibell^®^ size during the procedure. Choosing the correct size of Plastibell is crucial. Hollister, who invented Plastibell, advises that a too-small fit can cause tissue strangulation and necrosis, and that using one that is too large may result in too much foreskin being removed and penile denudation (8). The general practice is to choose the Plastibell size from visual estimation of the size of the glans after dorsal slit and adhesiolysis have been done. This may result in error in size selection. Also because the decision on the appropriate size is made during the procedure, it means that all sizes of Plastibell must have to be available in order to forestall a problem. This means cost of procuring all sizes at all time of the procedure. And when any particular size which is adjudged to be the best-fit is not available, it leads to choosing the second-best available. This is as any glans can be “forced” to accept the next bigger or smaller size. This will either predispose to risk of proximal migration, retention of the Plastibell ring, or denudation of penile skin post-circumcision (7). So we have problems of avoidable complications and increased cost arising from the choice of or the need to choose appropriate size of Plastibell for every circumcision on a child. There is therefore a need to find a way to make a decision on the best-fit or appropriate size of Plastibell to use for every child, before the procedure begins or at the planning stage.

The penile and glans size varies with each neonate. We may not know what determine the sizes at birth. But we do know that they increase with age. The penile sizes - length and diameter may affect the size of appropriate Plastibell device. As weight increases with age, there may be a chance that weight may affect size of penis and hence of the Plastibell. These are purely anthropometric measures.

This study was conceived to see if any correlation between the anthropometric parameters and the appropriate Plastibell size of a neonate. It also aims at determining whether these anthropometric parameters predict or help in determining, either singly or in combination, the choice of appropriate size of Plastibell ring for each neonatal circumcision.

## Methods

This was a cross-sectional study conducted over a 24-month period on all neonates who had Plastibell circumcision from November 2013 to December 2015. The study was conducted at Nnamdi Azikiwe University Teaching Hospital Nnewi, Anambra State, Nigeria. It was a single centre study. The study was carried out in neonates who had Plastibell^®^ circumcision. Approval was obtained from the Hospital Ethics Board (NAUTH/CS/66/VOL 5/01 & 82) for this study. Included in this study were all term male neonates who had Plastibell circumcision during that period. Excluded were any child with age above 28 days, and those with incomplete data.

Data were collected on a proforma. The proforma records of neonates who had Plastibell circumcision were retrieved. During the period of November 2013 to December 2015, the neonates who had Plastibell circumcision had their data collected in a proforma. They underwent the normal institutional procedure for neonatal circumcision with Plastibell ring under dorsal penile nerve block (DPNB) or eutectic mixture of local anaesthetics (EMLA) or oral ketamine as procedural anaesthesia / analgesia. Parameters collected were age in days, weight in Kg, penile length in cm and penile diameter in cm. The penile length was Stretched penile length (SPL). The measurement was taken by fully stretching the penis without causing discomfort. An assistant holds one end of the silk suture at the peno-pubic junction, and the circumciser draws it taut to the tip of the palpable glans. The estimated silk suture length is then applied to a ruler calibrated in centimetres. The penile diameter was estimated with the same silk suture just proximal to the glans. The glans was identified by palpation. This estimate was then applied on the same ruler calibrated in centimetres.

The procedures were carried out by one surgeon. The person that took measurement of penile lengths and diameters was not same as the surgeon. The surgeon made a choice of appropriate-sized Plastibell for each penis after adhesiolysis and dorsal slit stages by visual estimation as is the norm in the institution. All Plastibell sizes were available of at all times of the study period.

Data were keyed into SPSS ver. 25 and analysed. Analysis done were mean of anthropometric parameters; count of neonates in each Plastibell-size group; counts of anthropometric parameters in each Plastibell-size group; Pearson correlation of anthropometric parameters with themselves and Plastibell sizes; and logistic regression of parameters with relation to each Plastibell size. Significance was set at P≤0.05. Analysis was done with SPSS ver. 25

## Results

A total of 231 male neonates were recruited for the study but 224 with complete data were analysed. Their mean age was 15.6± 5.73 days. The mean weight was 3.7± 0.58 Kg. The mean penile length and diameter were 3.66 ± 0.58 cm and 3.79±0.64 cm, respectively (Table 1).

**Table 1:** Mean, anthropometry, distribution of Plastibell sizes and comparison of the parameters of the neonates on various plastibell sizes

Variables	Statistics	1.1	1.2	1.3	1.4	1.5	P-value	Total
No of neonates for each size	N (%)	7 (3.1)	50 (22.3)	120(53.6)	34 (15.2)	13 (5.8)	-	224 (100)
Age (days)	Min	9.0	7.0	4.0	8.0	11.0		4.0
	Max	23.0	28.0	28.0	28.0	28.0		28.0
	Mean(SD)	14.1±5.43	16.2±5.23	14.6±5.41	17.3±6.75	19.1±5.8	0.012*	15.58±5.73
Weight (kg)	Min	2.5	2.7	2.5	3.0	3.0		2.5
	Max	4.4	5.0	5.4	5.7	4.7		5.6
	Mean(SD)	3.2±0.69	3.5±0.48	3.7±0.50	3.7±0.50	3.9±0.54	0.001*	3.70 ±0.53
Penile length, PL	Min	2.5	2.0	2.0	2.5	4.0		2.0
(cm)	Max	3.8	4.5	5.5	4.5	5.0		5.5
	Mean(SD)	3.1±0.44	3.4±0.58	3.6±0.54	4.0±0.48	4.3±0.36	<0.001*	3.66±0.58
	Min	2.0	2.0	2.2	3.0	3.5		2.0
Penile diameter,	Max	4.0	4.5	6.0	5.0	5.2		6.0
PD (cm)	Mean(SD)	3.2±0.68	3.5±0.64	3.7±0.64	4.0±0.43	4.4±0.43	<0.001*	3.79 ±0.64

The range of Plastibell sizes used were 1.1 to 1.5, with sizes 1.3 accounting for the highest usage, 120 times (53.6%) and sizes 1.1 accounting for the least in usage 7 (3.1%) (Table 1). There is a consistent increase in the Plastibell sizes as the mean age, weight, penile length and penile diameter increases. The exception to this observation is with the mean age at Plastibell size 1.3 which is below the mean age for Plastibell size 1.2. These differences were statistically significant when subjected to ANOVA (see Table 1). There was a significant positive correlation between weight, penile length and penile diameter, on one hand and the Plastibell sizes used, on the other hand (Table 2). Though age correlates positively and significantly with weight and penile diameter, it does not correlate with sizes of Plastibell.

**Table 2:** Correlations of anthropometric parameters with Plastibell size

Variables		Age (days)	Weight (kg)	Penile length (cm)	Penile diameter(cm)	Plastibell size (N=224)
Age (days)	Pearson correlation (r)	-	0.218	0.112	0.137	0.124
	P value	-	0.001*	0.096	0.041*	0.063
Weight (kg)	Pearson correlation (r)	0.218	-	0.162	0.125	0.266
	P value	0.001*	-	0.015*	0.062	<0.001*
Stretched	Pearson	0.112	0.162	-	0.484	0.419
Penile length ,	correlation (r)					
SPL (cm)	P value	0.096	0.015*	-	<0.001*	<0.001*
Penile	Pearson	0.137	0.125	0.484	-	0.341
diameter, PD	correlation (r)					
(cm)	P value	0.041*	0.062	<0.001*	-	<0.001*

The anthropometric parameters were subjected to a multi-variate logistic regression to find out if any of them could be a determinant or predictor of Plastibell sizes to be used. Age has a weak capacity in determining the use of Plastibell size 1.3 (OR 1.076, 95% CI = 1.024 - 1.131; P=.004). Weight has 7x likelihood (OR 7.104; 95% CI 1.108 - 45.559; P = .039) and 2x likelihood (OR 2.044; 95% CI 1.054 - 3.963; P = .034) of predicting the appropriate use of Plastibell sizes 1.1 and 1.2, respectively. Penile length also predicted the use of Plastibell sizes 1.2 and 1.5. Penile diameter predicted the use of Plastibell size 1.5. (Table 3). No single anthropometry could predict the use of all Plastibell sizes.

**Table 3:** Multivariate logistic regression analysis of determinants of all Plastibell sizes

Variables	Plastibell sizes	Odd ratio	P value	95% CI
Age	1.1	0.994	.937	.862 – 1.147
	1.2	0.947	.065	.893– 1.003
	1.3	1.076	.004*	1.024 – 1.131
	1.4	.964	.281	.902 – 1.030
	1.5	.903	.073	.808 – 1.009
Weight	1.1	7.104	.039*	1.108 – 45.559
	1.2	2.044	.034*	1.054 – 3.963
	1.3	.767	.328	.452 – 1.304
	1.4	.517	.078	.248 – 1.078
	1.5	.846	.794	.241 – 2.969
Stretched Penile length, SPL	1.1	2.540	.240	.537 – 12.007
	1.2	2.176	.019*	1.136 – 4.136
	1.3	1.117	.684	.656 – 1.902
	1.4	.455	.059	.201 – 1.031
	1.5	.043	.001*	.007 - .260
Penile diameter, PD	1.1	1.992	.277	.575 – 6.902
	1.2	1.386	.260	.785 – 2.446
	1.3	1.029	.909	.633 – 1.672
	1.4	.567	.140	.267 – 1.204
	1.5	.266	.047*	.072 - .984

## Discussion

Circumcision is the commonest surgical procedure (1). Plastibell method is very widely accepted world-wide and is very common method for neonatal and infant circumcision in Nigeria (6,10,11). It is also prone to some complications that are partly or wholly a product of choosing a wrong-sized Plastibell such as proximal migration or retained Plastibell (11). It is therefore imperative that how to choose appropriate sized Plastibell is enhanced. The conventional way of choosing Plastibell size is by estimation of the circumference of the widest area of the exposed glans (12). This entails that the procedure is already underway before a choice can be made. This is froth with uncertainties and forces an availability of all Plastibell sizes before it is “safe” to start the procedure. Devising a way to determine the appropriate size of Plastibell before the procedure will help in improving the overall ease and safety of Plastibell circumcision. It is thought that the anthropometric characteristics will help in this determination, since the size of the glans is like every other human tissue/organ and varies with the overall growth of the person.

There were 224 neonates of which 120 of them used Plastibell size 1.3. This made Plastibell size 1.3, the most used, 53.6% in all. This is in line with the findings of Al-Marhoon MS et al[13], in which size 1.3 is the most commonly used size. Despite the increasing mean age being directly proportional to the Plastibell size used (except at size 1.3), it will be noted that there was a wide age range of neonates who could use Plastibell size 1.3. Likewise this age range was noted in all Plastibell sizes. The implication of this is that irrespective of age, most penile glans has about same size. This is explained by the fact that penile size is about a constant in infancy due to a steady state of serum testosterone required for penile growth (14,15). It becomes a disincentive for using age to determine the appropriate size of Plastibell in neonates.

The mean weight, penile length and penile diameter increases with the increasing Plastibell size in this study. These variations were statistically significant. And it was also reflected in the Pearson correlation, as they positively and significantly correlate with Plastibell^®^ sizes. While many studies have attempted to show how to choose Plastibell size, none related it to weight, penile length and penile diameter. However Nasir et al(16) directly measured the circumference of glans and used it to determine the diameter of glans which they equated to the Plastibell size (circumference). This study was done for infants and not only neonates. The positive correlation in this study offers a glimmer of hope that they could help predict the appropriate size of Plastibell. However it did not turn out so. No anthropometric parameter could strongly predict appropriate size; except weight for size 1.1 but it was not reflected in other sizes hence could not be applied. This shows that glans diameter varies widely from penile length, penile diameter and weight. Probably these were determined in-utero in no steady way. Hence similar glans diameter will have different penile length and penile diameter. The embryology and anatomy of the penis may also explain this variation, in which the glans seems to be a cap into which the corpora (forming the shaft) inserts (17).

This study was llimited by our inability to follow-up these neonates for a long period. We were unable to ascertain whether any complications like retention of the Plastibell and proximal migration of the Plastibell ring occurred with the neonates.

We conclude that the appropriate Plastibell size varies directly and correlates with the weight, penile length and penile diameter. One may be able to make a fair guess in the choice of appropriate Plastibell size by using these anthropometric parameters. However it will be necessary to develop an appropriate algorithm that can relate the anthropometries of each neonate with the glans diameter/circumference, since these anthropometries correlates positively with the glans circumference/diameter (which is equivalent to Plastibell size (17)). A more robust study and mathematical equation, therefore needed to be developed to help solve this problem. The end is to determine the appropriate Plastibell size before commencement of the procedure.
